# Racial disparities of Black Americans hospitalized for decompensated liver cirrhosis

**DOI:** 10.1186/s12876-020-01392-y

**Published:** 2020-07-29

**Authors:** Ted Spiewak, Amir Taefi, Shruti Patel, Chin-Shang Li, Eric Chak

**Affiliations:** 1 Department of Internal Medicine, UC Davis Medical Center, Sacramento, California USA; 2 Department of Gastroenterology and Hepatology, UC Davis Medical Center, 4150 V Street, PSSB 3500, Sacramento, CA 95817 USA; 3 School of Nursing, The State University of New York at Buffalo, Buffalo, New York USA

**Keywords:** African Americans, Liver cirrhosis, Health care disparities

## Abstract

**Background:**

Racial disparities have been reported in liver transplantation and chronic hepatitis C treatment outcomes. Determining causes of these disparities is important given the racially diverse American population and the economic burden associated with chronic liver disease.

**Methods:**

A retrospective study was performed among 463 patients diagnosed with cirrhosis admitted from (January 1, 2013 to January 1, 2018) to a tertiary care academic medical center. Patients were identified based on the International Classification of Diseases (ICD-10) for cirrhosis or its complications. Demographic information, laboratory data, medical comorbidities, insurance and adherence to cirrhosis quality care indicators were recorded to determine their relationship to readmission rates and other healthcare outcomes.

**Results:**

A total of 463 individual patients with cirrhosis were identified including Whites (*n* = 241), Hispanics (*n* = 106), Blacks (*n* = 50), Asian and Pacific Islander Americans (API, *n* = 27) and Other (*n* = 39). A significantly higher proportion of Blacks had Medicaid insurance compared to Whites (40% versus 20%, *p* = 0.0002) and Blacks had lower median income than Whites ($45,710 versus $54,844, *p* = 0.01). All groups received high quality cirrhosis care. Regarding healthcare outcomes, Black patients had the highest mean total hospital admissions (6.1 ± 6.3, *p* = 0.01) and the highest mean number of 30-day re-admissions (2.1 ± 3.7, *p* = 0.05) compared to all other racial groups. Multivariable proportional odds regression analysis showed that race was a statistically significant predictor of 90-day readmission (*p* = 0.03).

**Conclusions:**

Black Americans hospitalized for complications of cirrhosis may experience significant disparities in healthcare outcomes compared to Whites despite high quality cirrhosis care. Socioeconomic factors may contribute to these disparities.

## Background

The care of patients with chronic liver disease and cirrhosis is a costly endeavor for the United States healthcare system. The most recent estimates suggest that at least $2.5 billion yearly is spent on the care of these high-risk liver patients alone [1, 2]. Hospital readmissions among those with decompensated cirrhosis are a common and preventable occurrence with up to 53% of patients readmitted within 90 days [3, 4]. Due to the alarming cost of hospital readmissions, [5] interventions to reduce cirrhosis readmission have been developed to address this problem [6, 7].

According to the Centers for Disease Control and Prevention (CDC), health disparities are defined as preventable differences in the burden of disease, injury, violence, or opportunities to achieve optimal health that are experienced by socially disadvantaged populations. Racial and ethnic minorities have been shown to experience lower quality of health services compared to White Americans across a spectrum of chronic disease [8]. Racial disparities in liver transplantation [9, 10] and chronic hepatitis C treatment response have previously been reported [11].

Racial disparities in cirrhosis is an emerging area of research. It has previously been shown that non-White race has been associated with more frequent re-admission in patients with cirrhosis [12]. Further, an analysis of the Nationwide Inpatient Sample showed that Blacks and Hispanics were less likely to receive portosystemic shunt and liver transplantation compared to Whites and Blacks had higher in-hospital mortality than Whites [13]. More recently, in a large analysis of patients with cirrhosis across 4 American safety-net hospitals, no racial differences in mortality and 30-day were found [14]. Similarly, a nationwide sample showed increased unadjusted odds of mortality among Blacks, which dissipated after adjustment for potential confounders [15]. Thus, the effect of race on cirrhosis health outcomes is not well established.

Decreasing health disparities becomes increasingly important over time since racial and ethnic minorities may comprise over half of the United States population by 2050 [16]. Since death from cirrhosis or end stage liver disease is relatively common in United States [17], reducing cirrhosis-related health disparities will also serve to benefit the wellness of our population as a whole. The purpose of our study is to analyze the effect of race, income, and health insurance on cirrhosis-related outcomes such as receipt of quality cirrhosis care, hospital readmission, and mortality among patients hospitalized for decompensated cirrhosis.

## Methods

### Study design and patient population

We performed a retrospective study of all patients diagnosed with cirrhosis and admitted to UC Davis Medical Center (UCDMC) between January 1, 2013 and January 1, 2018, a tertiary care academic medical center. Any available data prior to January 1, 2013 was not analyzed to emphasize the most current data available. Medical chart review was performed of the UCDMC electronic medical records, with patients identified initially based on the International Classification of Diseases (ICD-10) for cirrhosis or its complications. On October 1, 2015, ICD-10 became available in the United States. At that time, our electronic health record system (Epic Systems, Verona, WI) automatically converted all existing ICD-9 codes to the equivalent ICD-10 codes. Thus, during data abstraction for this study, all codes had been converted to ICD-10 codes. The specific (ICD-10) codes used to determine cirrhosis and complications of cirrhosis included, alcoholic cirrhosis of the liver without ascites (K70.30), alcoholic cirrhosis of the liver with ascites (K70.31), unspecified cirrhosis of the liver (K74.60), other cirrhosis of the liver (K74.69) esophageal varices with bleeding (I85.01), gastric varices (I86.4), hepatic failure, unspecified with coma (K72.91), other ascites (R18.8) or spontaneous bacterial peritonitis (SBP) (K65.2). Demographic information, laboratory data, medical comorbidities, insurance, adherence to cirrhosis quality care indicators, 30 and 90-day readmission, and 30 and 90 day mortality were recorded for analysis. Median income was determined by zip code using a publically available online database (Income By Zip Code) of the American Community Survey 2017 5-year estimates. This study was approved by the Institutional Review Board at UCDMC.

For our study, we focused on the 3 major types of insurance in the United States: Medicare, Medicaid, and private insurance. Both Medicare and Medicaid are federal insurance programs. Medicare covers patients ages 65 and older (or ages less than 65 with a disability and dialysis patients). Medicaid covers low-income patients regardless of age. Lastly, private insurance is purchased by individuals or their employers. Thus, American patients with higher income are more likely to hold private insurance.

### Adherence to cirrhosis quality indicators

We determined adherence to cirrhosis quality indicators by assessing the following: receiving beta-blockers at discharge for secondary prophylaxis after being admitted for variceal bleed, receiving prophylactic antibiotics (IV ceftriaxone or equivalent) in the setting acute variceal hemorrhage, receiving diuretics at discharge for medium to large ascites in the absence of renal failure, receiving spontaneous bacterial peritonitis (SBP) prophylaxis at discharge with after diagnosis and treatment of SBP, receiving intravenous albumin as an inpatient in the setting acute SBP (1.5 g/kg IV on day 1, then 1 g/kg on day 3), receiving IV antibiotics (cefotaxime or equivalent) for treatment of SBP, and receiving lactulose and/or rifaximin for hepatic encephalopathy (HE). Patients who did not receive this level of care in any of these categories were counted as non-adherent for our analyses for each respective category.

### Statistical analysis

Descriptive data was reported as percentages, means ± SD and medians (with range and confidence interval when appropriate). For comparative analytics, we used Kruskal-Wallis test for continuous/numerical variables and Fisher’s exact test for categorical variables. Multivariable proportional odds regression analysis (hereafter called “multivariable regression analysis” unless otherwise specified) analysis was done to identify independent associations connected to racial disparities in cirrhosis related health care and their effect on readmissions and mortality. To create our multivariate regression models for 30 and 90-day readmissions and mortality, we adjusted for the following variables: ethnicity, MELD-Na, medical comorbidities (including diabetes, coronary artery disease, and chronic obstructive pulmonary disease), complications of cirrhosis (including hepatic encephalopathy, gastro-esophageal varices, ascites, and hepatocellular carcinoma), receipt of aforementioned quality cirrhosis care, English primary language, insurance status, and median income. A *p* value < 0.05 was considered significant.

## Results

A total of 463 individual patients with cirrhosis were identified including Whites (*n* = 241), Hispanics (*n* = 106), Blacks (*n* = 50), and Asian and Pacific Islander Americans (API, *n* = 27). The remaining 39 patients were categorized as Other. Mean age was 57.2 ± 10.8 years and the majority of the patients were male (58.1%). A significantly higher proportion of Blacks, Hispanics, and API had Medicaid compared to Whites (*p* = 0.0002). Blacks and Hispanics also had lower median income than Whites ($45,710 versus $54,844, *p* = 0.01) (Table 1). Further, Blacks and Hispanics had higher mean MELD-Sodium (MELD-Na) scores compared to Whites (28.7 ± 9.3 and 29.1 ± 8.5 versus 26.4 ± 9.5, *p* = 0.02) (Table 2).

**Table 1:** Baseline Demographics of Cirrhosis Patients Admitted to UC Davis Health System, January 2013 to January 2018

Characteristics	Whites (***N*** = 241)	Hispanics (***N*** = 106)	Blacks (***N*** = 50)	API (***N*** = 27)	Other (***N*** = 39)	***P*** value
Age (Mean ± SD)	58.1 ± 10.7	55.3 ± 11.3	57.0 ± 8.6	61.2 ± 14.5	54.5 ± 8.7	0.07
Male (%)	142 (58.9)	67 (63.2)	26 (52.0)	12 (44.4)	22 (56.4)	0.40
English Language Primary (%)	231 (95.9)	83 (78.3)	50 (100.0)	13 (48.2)	36 (92.3)	< 0.0001
Insurance (%)						0.0002
None	158 (34.13)	93 (38.59)	27 (25.47)	9 (18.00)	8 (29.63)	
VA Insurance	2 (0.43)	1 (0.41)	1 (0.94)	0 (0.00)	0 (0.00)	
Private	27 (11.2)	3 (2.8)	3 (6.0)	0 (0.0)	2 (5.1)	
Medicaid	48 (19.9)	44 (41.5)	20 (40.0)	11 (40.7)	8 (20.5)	
Medicare	72 (29.9)	31 (29.3)	18 (36.0)	8 (29.6)	8 (20.5)	
Median Income (minimum, maximum)	$54,488.5 ($0, $134,030)	$45,710.0 ($29,747, 115,600)	$45,710.0 ($29,747, $91,539)	$47,405.0 ($29,747, $10,2865)	$53,728.0 ($29,747, $102,865)	0.01

*API* Asian and Pacific Islander

**Table 2:** Mean Baseline Laboratory Values of Cirrhosis Patients Admitted to UC Davis Health System, January 2013 to January 2018

Characteristics	Whites (***N*** = 241)	Hispanics (***N*** = 106)	Blacks (***N*** = 50)	API (***N*** = 27)	Other (***N*** = 39)	***P*** value
Platelet ± SD	120.0 ± 75.1	104.1 ± 70.0	108.6 ± 60.9	114.1 ± 95.2	98.0 ± 51.4	0.19
Albumin ± SD	2.8 ± 0.8	2.6 ± 0.9	2.6 ± 0.8	2.6 ± 0.8	2.6 ± 0.7	0.04
Sodium ± SD	135.7 ± 5.4	135.9 ± 4.7	136.0 ± 5.2	137.7 ± 4.8	135.7 ± 5.0	0.39
Creatinine ± SD	1.6 ± 1.3	1.4 ± 1.9	2.5 ± 2.4	1.4 ± 1.0	1.4 ± 1.1	0.03
Total bilirubin ± SD	5.8 ± 9.3	5.4 ± 7.7	5.5 ± 7.7	4.2 ± 8.1	4.7 ± 7.8	0.63
INR ± SD	1.6 ± 0.8	1.5 ± 0.5	1.6 ± 0.6	1.4 ± 0.4	1.5 ± 0.4	0.60
MELD-Na ± SD	26.4 ± 9.5	29.1 ± 8.5	28.7 ± 9.3	24.2 ± 10.0	26.6 ± 9.3	0.02

*API* Asian and Pacific Islander, *INR* International normalized ratio, *MELD-Na* Model for End Stage Liver Disease-Sodium

Regarding medical co-morbidities, API (70.4%) and Blacks (44.0%) had significantly higher prevalence of diabetes mellitus (DM) compared to Whites (33.3%, *p* = 0.004). Blacks also had the highest prevalence of coronary artery disease (54.0%, *p* = 0.0004) and chronic hepatitis C (38.0%, *p* <  0.0001) compared to other racial groups (Table 3).

**Table 3:** Baseline Co-morbidities and Liver Decompensations of Cirrhosis Patients Admitted to UC Davis Health System, January 2013 to January 2018

Characteristics	Whites (***N*** = 241)	Hispanics (***N*** = 106)	Blacks (***N*** = 50)	API (***N*** = 27)	Other (***N*** = 39)	***P*** value
Diabetes N (%)	80 (33.3)	40 (37.7)	22 (44.0)	19 (70.4)	17 (44.7)	0.004
Coronary artery disease (%)	65 (27.0)	25 (23.6)	27 (54.0)	6 (22.2)	5 (13.5)	0.0004
COPD (%)	50 (20.9)	14 (13.2)	13 (26.0)	4 (15.4)	4 (10.5)	0.17
Smoking (%)	165 (69.0)	67 (64.4)	37 (74.0)	15 (55.6)	29 (76.3)	0.34
Hepatic encephalopathy (%)	151 (62.9)	70 (66.0)	33 (66.0)	17 (63.0)	31 (79.5)	0.38
Gastro-esophageal varices (%)	96 (42.3)	61 (61.6)	20 (41.7)	14 (53.9)	19 (50.0)	0.02
Ascites (%)	163 (67.9)	65 (61.3)	30 (61.2)	17 (63.0)	25 (64.1)	0.74
Liver cancer (%)	2 (0.8)	1 (1.0)	1 (2.0)	2 (7.4)	1 (2.6)	0.09
Etiology of Liver Disease						< 0.0001
HCV (%)	60 (25.0)	26 (24.5)	19 (38.0)	6 (22.2)	9 (23.1)	
Alcohol (%)	82 (34.2)	46 (43.4)	15 (30.0)	3 (11.1)	15 (38.5)	
HCV + Alcohol (%)	36 (15.0)	15 (14.2)	5 (10.0)	0 (0.0)	7 (18.0)	
NASH (%)	24 (10.0)	2 (1.9)	0 (0.0)	5 (18.5)	4 (10.3)	
HBV (%)	3 (1.3)	0 (0.0)	1 (2.0)	7 (25.9)	0 (0.0)	
Autoimmune, PBC, PSC (%)	7 (2.9)	4 (3.8)	1 (2.0)	0 (0.0)	0 (0.0)	
Unknown, other (%)	28 (11.7)	13 (12.3)	9 (18.0)	6 (22.2)	4 (10.3)	

*API* Asian and Pacific Islander, *COPD* Chronic obstructive pulmonary disease, *HCV* Hepatitis C Virus, *NASH* non-alcoholic steatohepatitis, *PBC* primary biliary cholangitis, *PSC* primary sclerosing cholangitis

**Fig. 1:**
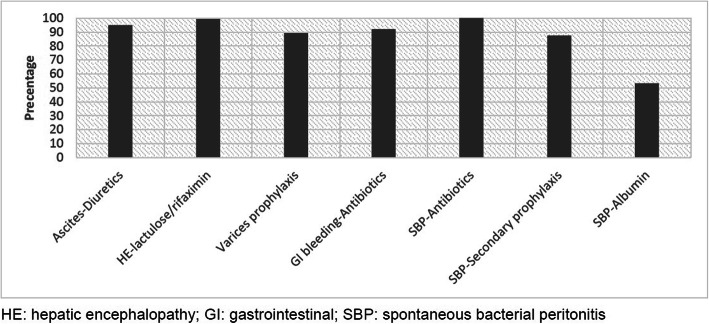
Baseline Adherence to Cirrhosis Quality Indicators at UC Davis Medical Center, January 2013–January 2018

Adherence to most cirrhosis quality care indicators ranged from 87 to 100% (Fig. 1) suggesting high quality inpatient cirrhosis care. The exception was that only 53.2% received the proper dosing of albumin in the setting of SBP. No racial disparities regarding receipt of quality cirrhosis care were identified.

Regarding healthcare outcomes, Black patients had the highest mean total hospital admissions (6.1 ± 6.3, *p* = 0.01), highest mean number of 30-day re-admissions (2.1 ± 3.7, *p* = 0.05). Blacks also trended towards the highest mean number of 90-day readmissions (3.7 ± 5.5, *P* = 0.10) and highest mean total hospital days (46.3 ± 54.5, *p* = 0.07) (Table 4).

**Table 4:** Healthcare-Related Outcomes of Cirrhosis Patients Admitted to UC Davis Health System, January 2013 to January 2018

Characteristics	Whites (***N*** = 241)	Hispanics (***N*** = 106)	Blacks (***N*** = 50)	API (***N*** = 27)	Other (***N*** = 39)	***P*** value
Mean Total Hospital Admissions	3.0 ± 3.0	3.4 ± 3.8	6.1 ± 6.3	4.5 ± 4.7	2.4 ± 1.8	0.01
Mean Total Hospital Days	22.0 ± 23.2	22.2 ± 23.6	46.3 ± 54.5	22.8 ± 21.7	18.0 ± 20.0	0.07
Mean Length of Hospital Stay (Days)	7.6 ± 6.4	7.3 ± 5.7	7.7 ± 6.2	5.3 ± 3.1	7.9 ± 7.4	0.56
Mean 30-day Readmission	0.7 ± 1.5	1.0 ± 2.2	2.1 ± 3.7	1.0 ± 1.5	0.5 ± 0.7	0.05
Mean 90-day Readmission	1.4 ± 2.4	1.5 ± 3.4	3.7 ± 5.5	1.9 ± 2.9	0.9 ± 1.2	0.10
30-day mortality (%)	60 (24.9)	23 (21.7)	12 (24.0)	4 (14.8)	9 (23.1)	0.85
90-day mortality (%)	7 (2.9)	6 (5.7)	3 (6.0)	0 (0.0)	2 (5.1)	0.44
Liver Transplanted (%)	9 (3.7)	6 (5.7)	3 (6.0)	0 (0.0)	3 (7.7)	0.52

*API* Asian and Pacific Islander

Because Black patients had statistically higher rates of CAD and the highest rates of COPD compared to other races analyzed, this may have confounded the relationship between Black race and hospital readmission. Therefore, multivariable regression analysis was performed to determine if race was an independent predictor of 30 and 90-day hospital readmission (Tables 5 and 6). When compared to Whites, Blacks had higher odds of 30-day hospital readmission OR = 2.13, 95% CI (1.14–3.95). Coronary artery disease (CAD), chronic obstructive pulmonary disease (COPD), hepatic encephalopathy (HE), and presence of ascites were all found to be independently associated with 30-day readmission on multivariable regression analysis. Insurance status was also found to be predictive of 30-day readmission. The odds ratio of 30-day readmission for no insurance versus private insurance was OR = 0.45, 95% CI (0.21–0.99) suggesting that no insurance was protective against 30-day readmission (Table 5).

**Table 5:** Multivariable Proportional Odds Regression Analysis of Factors Associated with 30-Day Readmissions at UC Davis Health System, January 2013 to January 2018

Characteristic	Odds ratio	95% CI	***P***-value
**Race**			0.21
Black versus White	2.13	1.14–3.95	0.02
Black versus API	1.94	0.72–5.24	0.19
**Co-morbidities**			
Coronary artery disease	1.76	1.10–2.80	0.02
COPD	1.87	1.15–3.05	0.01
Hepatic encephalopathy	2.42	1.53–3.85	0.00002
Diabetes	1.39	0.90–2.15	0.13
Ascites	1.96	1.23–3.11	0.004
**Insurance Status**			0.005
None versus Private	0.45	0.21–0.99	0.046
None versus Medicaid	0.40	0.23–0.67	0.0006
Medicaid versus Private	1.15	0.53–2.47	0.72
Medicaid versus Medicare	1.35	0.82–2.24	0.24

*API* Asian and Pacific Islander, *COPD* chronic obstructive pulmonary disease, *CI* confidence interval, *NS* not significant

**Table 6:** Multivariable Proportional Odds Regression Analysis of Factors Associated with 90-Day Readmissions at UC Davis Health System, January 2013 to January 2018

Characteristic	Odds Ratio	95% CI	***P***-value
**Race**			0.03
Black versus White	4.69	1.57–13.95	0.006
Black versus API	8.45	1.90–37.60	0.005
**Co-morbidities**			
Coronary artery disease	1.25	0.54–2.87	0.60
COPD	1.54	0.67–3.56	0.31
Hepatic encephalopathy	4.74	1.93–11.63	0.0007
Diabetes	2.07	1.01–4.22	0.047
Ascites	3.91	1.61–9.52	0.003
**Insurance Status**			0.0001
None versus Private	0.40	0.11–1.42	0.35
None versus Medicaid	0.15	0.06–0.39	< 0.0001
Medicaid versus Private	2.57	0.78–8.52	0.12
Medicaid versus Medicare	4.72	2.11–10.6	0.0002

*API* Asian and Pacific Islander, *COPD* chronic obstructive pulmonary disease, *CI* confidence interval, *NS* not significant

As shown in Table 6, race was a statistically significant predictor of 90-day readmission (*p* = 0.03). The odds ratio of 90-day readmission for Black versus White patients was OR = 4.69, 95% CI (1.57, 13.95). Insurance status was also a statistically significant predictor of 90-day readmission (*p* = 0.0001). The odds ratio of 90-day readmission for Medicaid insurance versus Medicare insurance was OR = 4.72, 95% CI (2.11–10.6).

Severity of cirrhosis was also found to be an important independent predictor of 30-day mortality in our cohort. Multivariable binary logistic regression analysis showed a statistically significant association between MELD-Na and 30-day mortality (OR = 1.06, 95% CI (1.03, 1.09), *p* <  0.0001. We did not, however, find a statistically significant association between MELD-Na and 90-day mortality OR = 1.02, 95% CI (0.972, 1.077), *p* = 0.07.

## Discussion

In this retrospective cohort study, Black patients admitted for decompensated liver cirrhosis had the highest mean total hospital admissions and mean number of 30-day readmissions compared to other racial groups. Black patients also trended towards the highest mean number of hospital days and 90-day readmissions, but these did not reach statistical significance. The disparities that were found may be the result of differences in socioeconomic factors such as insurance status. We found that Black patients were more likely to have Medicaid insurance compared to other racial groups at baseline. On multivariable regression analysis, Black race conferred a 2-fold increased odds in 30-day readmission and a 4-fold increased odds in 90-day readmission compared to White race. Further, multivariable regression analysis also showed that Medicaid insurance was found to confer a 4-fold increased odds of 90-day readmission compared to Medicare insurance. Interestingly, lack of insurance was associated with lower odds of readmission when compared to private and Medicaid insurance. One explanation for this could be that patients with no insurance also had other socioeconomic disparities (such as lack of transportation to the hospital) that prevented them from being readmitted.

Racial disparities in liver disease may result from a combination of biological, socioeconomic, and cultural factors [18]. After introduction of the MELD score, racial disparities have appeared to decrease regarding receipt of cadaveric liver transplantation [19]. However recent studies have noted that Blacks were less likely than Whites to receive living donor liver transplantation [10] and experience higher rates of liver transplant graft failure compared to Whites [9]. With regard to cirrhosis care, non-White race was independently shown to be associated with a 2-fold increase odds of readmission [12]. In a recent study of patients with hepatocellular carcinoma, Blacks were found to have had a significantly higher mortality compared to Whites after adjusting for tumor stage, liver function, receipt of HCC treatment, and insurance status [20]. Our findings add to the growing body of literature showing that Blacks may experience chronic liver disease disparity.

Differences seen in the receipt and quality of healthcare pertaining to those with liver disease may also be related to health insurance status and lower socioeconomic status. Significant disparities in access to treatment of chronic HCV infection since the approval of direct acting antiviral (DAA) agents, have been reported [21]. Medicaid has been associated with a higher proportion of patients receiving absolute denials [22] and significantly lower odds of treatment [23] than those covered by Medicare and those who are commercially insured. Younossi ZM, et al., reported insurance-specific disparities after analyzing the non-start rates of patients who were prescribed sofosbuvir-based regimens. Non-start rates were the highest in Medicaid-covered patients at (35%), as compared to Medicare (2%) and commercial insurances (6%). This study also showed that those with commercial coverage were 6.5 times as likely to start sofosbuvir based therapy compared to patients with Medicaid [24]. Medicaid has also been reported to be associated with higher Model for End-Stage Liver Disease (MELD) scores at transplant registration and also associated with worse post-transplant outcomes [25].

Adherence to evidence based cirrhosis care has been previously been described as suboptimal [26, 27]. In a study of Veterans Affairs patients with cirrhosis complicated by ascites, only 33.2% of patients receiving all recommended care [28]. Similar findings of suboptimal cirrhosis care have been reported in the setting of variceal bleeding, screening for varices, and HCC surveillance with ultrasound [29–31]. Compared to these previous studies, adherence to evidence based cirrhosis care at our institution was improved with 87–100% adherence to most quality indicators which may reflect increased awareness of cirrhosis care in the current era. No racial disparities in receipt of quality cirrhosis care were identified in our study, which suggests that racial disparities in cirrhosis outcomes occurred despite high quality cirrhosis care.

Chronic comorbid conditions [32] and complications of liver cirrhosis [33] are both predictors of hospital readmission and poor healthcare outcomes. In patients with cirrhosis, hepatic encephalopathy (HE) and ascites are most strongly associated with readmission within 30 or 90 days [33–35]. Our study’s findings align with what is reported in the literature with respect to these well-established risk factors of readmission following a hospitalization for decompensated liver cirrhosis. On multivariable analysis, the presence of HE conferred a 2-fold increased odds in 30-day readmission and a 4-fold increased odds in 90-day readmission. The presence of ascites also nearly conferred a 2-fold increased odds in 30-day readmission and a 4-fold increased odds in 90-day readmission. MELD-Na was associated with an increased odds of 30-day mortality on multivariate binary logistic regression analysis. Further, CAD and DM conferred nearly a 2-fold increased odds in 30-day and 90-day readmission, respectively.

Limitations of our study are typical for retrospective design. The categorization of patients as being diagnosed with cirrhosis and its complications by ICD-10 codes and their assignment into racial groups was dependent on accuracy of this information as recorded in the electronic health record. Further, hospital readmission, mortality, and liver transplantation, which occurred outside of our health system, would not have been captured when data was abstracted. Lastly, patients were identified only by ICD-10 code. Thus, we were able to determine the presence of cirrhosis complications such as ascites and hepatic encephalopathy, but we were unable to determine Child-Pugh scores since severity of ascites and hepatic encephalopathy was not captured. Therefore, potential associations between healthcare outcomes and Child-Pugh scores would have been missed.

## Conclusions

We found racial disparities in Black Americans hospitalized for decompensated liver cirrhosis. Black race was independently associated with increased odds of hospital readmission. At baseline, Medicaid insurance was more prevalent in Black patients and was independently associated with higher odds of readmission. This study highlights the need for further etiologic studies on racial disparities in cirrhosis care specifically to identify actionable intervention targets in order to reduce these disparities seen in the Black community.

## Supplementary information

**Additional file 1.**

## Data Availability

All data generated or analyzed during this study are included in this published article as a supplemental file.

## Abbreviations

API: Asian and Pacific Islander
CAD: Coronary artery disease
CI: Confidence interval
COPD: Chronic obstructive pulmonary disease
DM: Diabetes mellitus
DAA: Direct acting antiviral
GI: Gastrointestinal
HE: Hepatic encephalopathy
ICD: International Classification of Diseases
MELD: Model for end-stage liver disease
SBP: Spontaneous bacterial peritonitis
UCDMC: UC Davis Medical Center
